# An Automatic Gait Analysis Pipeline for Wearable Sensors: A Pilot Study in Parkinson’s Disease

**DOI:** 10.3390/s21248286

**Published:** 2021-12-11

**Authors:** Luis R. Peraza, Kirsi M. Kinnunen, Roisin McNaney, Ian J. Craddock, Alan L. Whone, Catherine Morgan, Richard Joules, Robin Wolz

**Affiliations:** 1 IXICO, London EC1A 9PN, UK; luis.peraza@ixico.com (L.R.P.); kirsi.kinnunen@ixico.com (K.M.K.); richard.joules@ixico.com (R.J.); robin.wolz@ixico.com (R.W.); 2 Department of Human Centred Computing, Monash University, Clayton, VIC 3800, Australia; roisin.mcnaney@monash.edu; 3 Electrical and Electronic Engineering, School of Computer Science, University of Bristol, Bristol BS8 1QU, UK; ian.craddock@bristol.ac.uk; 4 Translational Health Sciences, University of Bristol Medical School, Bristol BS8 1QU, UK; alan.whone@bristol.ac.uk; 5 Movement Disorders Group, North Bristol NHS Trust, Westbury on Trym, Bristol BS10 5NB, UK; 6 Department of Computing, Imperial College London, London SW7 2AZ, UK

**Keywords:** accelerometry, deep learning, free living, step length, initial contact, toe-off

## Abstract

The use of wearable sensors allows continuous recordings of physical activity from participants in free-living or at-home clinical studies. The large amount of data collected demands automatic analysis pipelines to extract gait parameters that can be used as clinical endpoints. We introduce a deep learning-based automatic pipeline for wearables that processes tri-axial accelerometry data and extracts gait events—bout segmentation, initial contact (IC), and final contact (FC)—from a single sensor located at either the lower back (near L5), shin or wrist. The gait events detected are posteriorly used for gait parameter estimation, such as step time, length, and symmetry. We report results from a leave-one-subject-out (LOSO) validation on a pilot study dataset of five participants clinically diagnosed with Parkinson’s disease (PD) and six healthy controls (HC). Participants wore sensors at three body locations and walked on a pressure-sensing walkway to obtain reference gait data. Mean absolute errors (MAE) for the IC events ranged from 22.82 to 33.09 milliseconds (msecs) for the lower back sensor while for the shin and wrist sensors, MAE ranges were 28.56–64.66 and 40.19–72.50 msecs, respectively. For the FC-event detection, MAE ranges were 29.06–48.42, 40.19–72.70 and 36.06–60.18 msecs for the lumbar, wrist and shin sensors, respectively. Intraclass correlation coefficients, ICC(2,k), between the estimated parameters and the reference data resulted in good-to-excellent agreement (ICC ≥ 0.84) for the lumbar and shin sensors, excluding the double support time (ICC = 0.37 lumbar and 0.38 shin) and swing time (ICC = 0.55 lumbar and 0.59 shin). The wrist sensor also showed good agreements, but the ICCs were lower overall than for the other two sensors. Our proposed analysis pipeline has the potential to extract up to 100 gait-related parameters, and we expect our contribution will further support developments in the fields of wearable sensors, digital health, and remote monitoring in clinical trials.

## 1. Introduction

The analysis of gait is highly informative in the clinical evaluation of patients with diseases involving movement abnormalities. Instrumented and objective methods to study gait abnormalities could help to improve diagnosis, stratify or enrich study populations, track disease progression, and assess the efficacy of new treatments. The gold standard for instrumented gait analysis is to use pressure-sensing platforms or walkways, which consist of an array of sensors that can capture individual foot-strikes while a participant performs a series of directed tasks. Pressure-sensing walkways can capture the initial contact (IC) and final contact (FC) events for each foot-strike and estimate gait parameters such as step length, step time, and symmetry. These metrics are then incorporated into the diagnostic or follow-up clinical assessment of patient with movement disorders [1].

Despite the proven utility of gait analysis in clinical settings, the use of pressure-sensing walkways can be prohibitive in other scenarios such as free-living or at-home studies; pressure-sensing walkways are expensive and the hardware requires specialist setup, which has led to most gait-related studies to be completed in specialised gait laboratories within clinics or hospitals. The increasing need for gait research in free-living settings has been driven by discrepancies between patients’ higher performance on gait tasks when screened in a clinical setting and their performance at home. This is called the Hawthorne effect [2]: patients tend to focus more on their assigned tasks while being observed by their physician, which leads to gait scores that do not reflect their disease stage or condition [3,4,5]. Additionally, in-clinic assessments offer only a snapshot of a patient’s condition. For instance, gait in PD is assessed in clinical settings using the UPDRS and the timed-up-and-go (TUG) tests; however, these tests only observe the status of patients during the test but are not able to assess gait during the rest of the day, or days before and after the tests. Gait performance in people diagnosed with PD can vary across several days [6], and technologies such as wearable sensors applied to free-living gait analysis can offer important insights into the patient’s daily symptomatic burden and variability [7].

Research studies have used cameras attached to the participant or within the participant’s home and/or have synchronised pressure-sensor insoles to assess free-living gait. This setup, the use of cameras or/and insoles, is currently widely used as ground truth for evaluating algorithms for inertial sensors in free-living gait research [8,9]. However, the use of cameras or insoles that need to be fitted inside shoes increases participant burden, and has similar problems as pressure-sensing walkways in terms of availability and potentially high cost of the required hardware [10].

The use of wearable sensors to assess free-living activity is increasingly attractive as these devices overcome many of the above-mentioned limitations. Technological advances have made wearable sensors lightweight, relatively inexpensive, with a battery life of up to a month, and with suitable acquisition rate for gait analysis (≥50 Hz, depending on the make and number of sensors that are “on”) [11]. Inertial measurement units (IMUs) are a type of wearable sensor commonly used for gait analysis, and which are equipped as a minimum with a tri-axial accelerometer, with often a gyroscope and a magnetometer additionally included. For gait analysis, the accelerometer and gyroscope within an IMU can be used with data fusion techniques to extract gait parameters. A gyroscope, however, consumes on average five times more energy than an accelerometer, and this can reduce the battery life of a sensor device from several weeks to a few days [12]. Depending on the application, some device manufacturers disable the IMU’s gyroscopes to allow for a longer battery life when long-term continuous data acquisition is required [12].

Several algorithms have been developed to extract gait measurements from IMU data, most of the developments use both gyroscopes and accelerometers for gait phase segmentation or detection of IC/FC events. There is, as expected, a trade-off in using/not using the gyroscope; algorithms that include gyroscope data report better performance than those that use accelerometry data only, at the cost of battery life. McCamley et al. reported a method to detect IC/FC events from vertical accelerometry acquired with a sensor attached on the lower back (near L5 lumbar vertebrae) [13]. The method is based on continuous wavelet transformations (CWT) and was further validated by Godfrey and colleagues [14,15], who also implemented the inverted pendulum model to estimate step length [16]. In the same fashion, but using gyroscope data, Greene et al. developed an adaptive algorithm for gait event estimation with sensors placed on the shanks. The authors reported good agreement between the sensor results and the pressure-sensing reference system for slow and normal gait speeds [17]. Trojaniello et al. also reported a double shank sensor-based algorithm that uses gyroscope data to detect IC/FC events and accelerometry to estimate step and stride lengths. The reported algorithm was shown to be robust and was validated with a large gait database that included patients with varied motor disorders [18]. 

Recent developments in artificial intelligence (AI) have allowed the expansion of applied AI in digital health, including the analysis of data from wearable sensors. The dominant deep neural network architectures for sensor data are long short-term memory (LSTM) units and convolutional neural networks (CNNs). In a recent report, Kreuzer and Munz implemented a combined CNN-LSTM network to perform gait phase segmentation using data from 11 IMUs attached to the foot, tibia, thigh, hip, and both arms. The authors used data from the gyroscope, accelerometry and barometer from all sensors as input to their neural network [19]. Yan et al. implemented a voting-weighted deep neural network (DNN) that was trained using tri-axial accelerometry data from three sensors located at the foot, thigh, and calf [20], and in a recent publication, Sarshar et al. implemented a LSTM-DNN using accelerometry, gyroscope and magnetometer data from two sensors attached to the participants’ shins [21]. One advantage of AI-based methods is that data fusion from multiple sensors is natural for DNN algorithms, and this technology can even obtain good results with raw signals as input—if provided with enough data—instead of designed features, which require data expertise in the field of gait analysis.

Although some investigations use multiple sensors to analyse gait data, in a free-living application multiple sensors will result in discomfort for study participants who may need to wear them 24/7, and this discomfort may prove intolerable in long-term clinical studies [22]. In this investigation, we report a deep learning-based analysis pipeline for wearable sensors. We focused our development on free-living and at-home gait assessment, aiming for continuous acquisition of gait data in long term studies of clinical populations. The pipeline accepts as input tri-axial accelerometry data from a single wearable sensor attached to either the lower back, shin, or wrist. We also report results of the pipeline’s performance on gait data acquired from a group of older adults with and without Parkinson’s disease.

## 2. Materials and Methods

### 2.1. Research Setting

All data were acquired within a two-bedroom terraced house located in Bristol, UK, named the SPHERE house. This fully furnished and highly instrumented “living lab” provides a naturalistic setting within which participants can freely live whilst being continuously sensed by ambient devices. The SPHERE house is part of the Sensor Platform for Healthcare in Residential Environment (SPHERE) project at the University of Bristol [23]. The study was conducted according to the guidelines of the Declaration of Helsinki and approved by the Ethics Committee of University of Bristol. 

### 2.2. Participants and Data Acquisition

Recruitment for this pilot study was through The Cure Parkinson Trust charity. The participants were six adults with a clinical diagnosis of Parkinson’s disease (PD) and six healthy controls (HC), who were spouses of the participants with PD. All participants provided informed consent, and, to ensure participant safety, had to be living independently. Accelerometry data were collected from all 12 participants. They wore four wearable sensors: one AX3 (axivity.com) on one wrist and one GENEActiv (activinsights.com) on the other wrist; participants with PD wore the AX3 device on the side more affected by bradykinesia, while for the HC participants the side of each device was randomised. One AX3 sensor was attached with hypoallergenic tape to the lower back (approximately on L5). The fourth device was a GENEActiv sensor strapped around the shin on the side more affected by bradykinesia in PD participants; in the HC group, the side was again randomised. Positioning of all sensors on a participant was implemented according to manufacturers’ recommendations (see Supplementary Material). Sensors were set at 100 Hz sampling rate and −/+8 g range for the accelerometers. 

In order to collect reference gait data, the participants were instructed to walk on a pressure-sensing walkway, Zeno Walkway system, by ProtoKinetics (www.protokinetics.com, accessed on 7 December 2021), which was customised to 83 cm width and 460 cm length to fit the designated floor space in the SPHERE House. The sampling rate was 120 Hz.

To enable offline synchronisation of the sensor devices and the walkway, markers in the recorded data were generated by mechanically tapping the devices on the sensing platform five times. Participants in the study performed four walking tasks on the walkway: slow gait, normal gait, fast gait, and the TUG task, where participants sat on a chair placed at the end of the walkway, stood up and walked to the other end of the walkway (at normal speed) before returning to the chair to sit down again. For the walking tasks, participants walked back and forth three to four times along the sensing walkway at each of the three speeds. Tasks were performed at the participant’s self-determined safe speed while imagining to be in different scenarios, e.g., for the slow gait task, participants were advised to imagine window shopping while for the fast gait participants were advised to imagine they needed to catch a bus.

### 2.3. Data Processing

Accelerometry datasets were downloaded from the sensor devices using the manufacturers’ software. Signal processing was implemented with in-house software based on Python’s library Scipy [24]. Data were synchronised between device recordings using markers introduced during device setup. AX3 datasets were resampled to 100 Hz using spline interpolation [25], and GENEActiv datasets were not resampled. Tri-axial accelerometry time series were filtered with a 5th order band-pass (0.5–20 Hz) Bessel filter with zero-phase treatment. For all sensor locations (shin, wrist, lumbar), the accelerometry data were re-labelled from the sensor’s x,y,z axes to the participant’s vertical (VT), medial-lateral (ML), and anterior-posterior (AP) orientations.

### 2.4. U-Net CNN for Gait Event Detection

**Figure 1:**
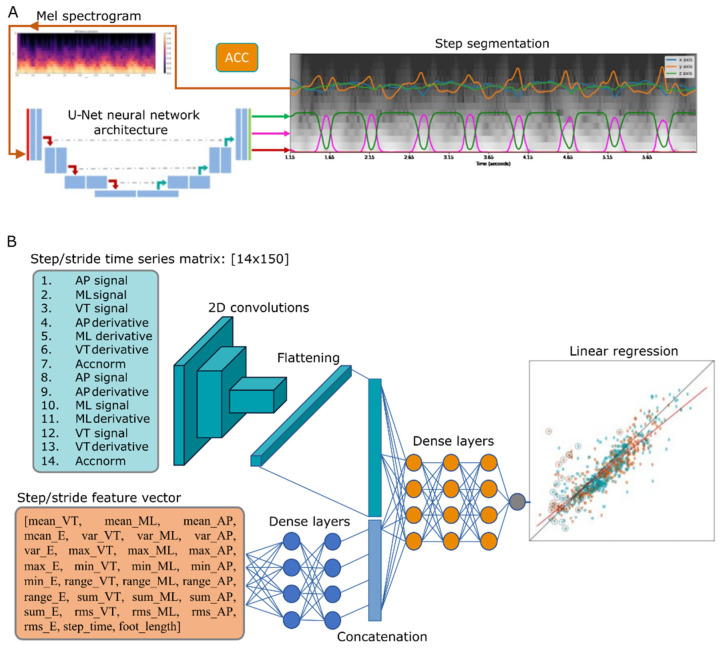
DNN models for gait event detection and step/stride length estimation. (**A**) Gait event detection model based on U-Nets. (**B**) Step/stride length estimation DNN models.

To detect IC, FC, and bout segments, we opted for a CNN with a U-Net architecture [26]. The U-Net CNN was originally designed for semantic segmentation in medical imaging and not for time series. Hence, we transformed the accelerometry time series to mel spectrograms using Librosa [27]. Mel spectrograms segments of 512 samples (or 5.12 s) of pre-processed actigraphy were obtained with an FFT-window of 32, 16 mel levels and hop length = 2; an example of mel spectrogram is shown in Figure 1A. The mel-transformed tri-axial accelerometry segments were then arranged in 3D arrays of size 16 × 256 × 3, similar to RGB images, where the depth dimension corresponded to the VT, ML and AP orientations. Finally, the mel-spectrogram images were normalised with an arbitrary constant (C = 80), to have values between 0 and 1 as inputs to the U-Net. 

Due to the small database in this investigation, we fine-tuned previously fully trained models with the same architecture, that detect heel-strikes from wrist-, lumbar- and shin-located sensors [28], one U-Net model per body location. In brief, these previously trained models were developed within an internal multi-activity investigation at IXICO, where 30 healthy adult participants were recruited. Four sensors were attached to their wrists, lower back, and shin, and participants were asked to walk eight times back and forth along a corridor and to perform multiple tasks: sitting still, pronation-supination, drawing a star, and a front-arm raise. Heel-strike events were detected with an in-house adaptive step detection algorithm applied to the lumbar sensor to obtain training labels [29]. 

For the training datasets in the current investigation, time series were divided in 512-sample segments with 128-sample hops, i.e., 75% overlapped segments. Label mask vectors were created for walking, FC, and IC events; the latter with a mask of ones that spanned 80 milliseconds (msecs) centred on the event, and according to the markers extracted from the sensing walkway. A schematic diagram of the U-Net learning procedure and data feeding is shown in Figure 1A. 

The U-Net was designed on TensorFlow-Keras (version 2.4) [30], with Adam optimiser, learning rate = 0.0001—one tenth of the original learning rate in [28], and a binary cross-entropy loss function. The fine tuning was implemented within the validation procedure explained in Section 2.7.

### 2.5. Step/Stride Length DNN Model

To estimate step/stride length, we opted for a deep learning approach, where designed features and processed time series are fed to a DNN, as shown in Figure 1B. Two DNN models were trained, one for step length and another for stride length. The network architecture comprises two input branches: a CNN and a two-layer DNN. The approach of fusing features and time series with DNNs has been proven successful in previous applications [31]. 

Using the IC label markers from the sensing walkway, accelerometry time series for individual steps and strides are extracted. For the CNN branch, the input matrix was built by interleaving the processed VT, ML and AP time series, their derivatives, and the Euclidian norm $Acc\;norm\left(t\right)=\sqrt{VT{\left(t\right)}^{2}+ML{\left(t\right)}^{2}+AP{\left(t\right)}^{2}}$ into a 14-row matrix as shown in Figure 1B. Each time series was resampled to a fixed length of 150 samples for steps and 300 samples for strides, in order to obtain fixed size input matrices for the CNN model, e.g., 14 × 150 and 14 × 300 matrices. As a final step, matrices were divided by an arbitrary constant (C = 8, same as the −/+8 g sensor range) to normalise input values in the [–1,1] range. 

The dense layer branch input was built with a feature vector extracted from step/stride time series. Features comprise the mean, variance, root mean square (RMS), range, the sum of all values, the step time in seconds and foot size in centimetres (divided by a constant 30) [32,33]. The foot size was estimated from the sensing walkway data and used as a proxy for the participant’s height since this latter demographic variable was not recorded in the study. The outputs of both branches are then concatenated in a single vector and fed to a three-layer DNN and a final perceptron with no activation function for linear regression. The entire step database comprised 725 steps, and we took as ground truth the absolute step length (distance between consecutive ICs) and the stride length (distance between consecutive ICs of the same foot) as estimated from the ProtoKinetics Movement Analysis Software (PKMAS). The target lengths were fed to the algorithm in cm, following division by a constant value of 100 and 200 for steps and strides, respectively.

The step and stride length DNN models were designed on TensorFlow-Keras (version 2.4) [30] and trained with Adam optimisation (learning rate = 0.001) and mean absolute error as loss function. For the estimation of step/stride length, we only used data from the lumbar sensor since preliminary results from the wrist and shin were unsatisfactory. The cross-validation strategy for the models is explained in Section 2.7.

### 2.6. Pipeline Design

**Figure 2:**
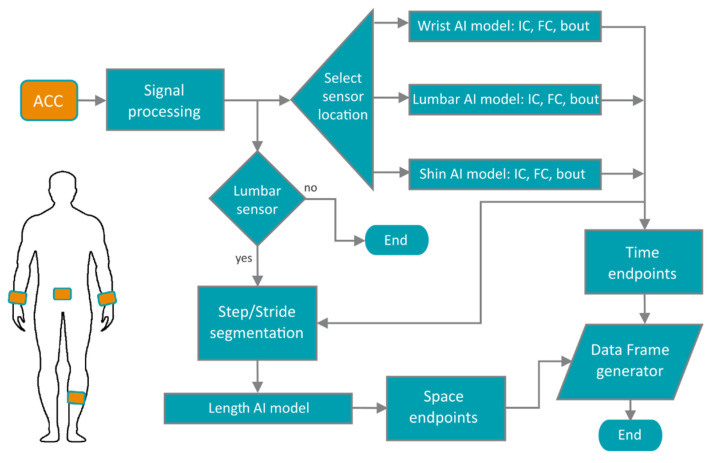
Gait analysis pipeline. The pipeline accepts sensors from three body locations: wrist, lumbar or shin. Then, accelerometry signals are extracted, preprocessed and a location-specific model is loaded for detection of gait events. If a lumbar sensor is inputted, models for step/stride length estimation are also loaded. As final step, the pipeline generates a CSV file, from a Pandas dataframe, with the results from the gait analysis.

The analysis pipeline is shown as a schematic in Figure 2. The pipeline receives a raw file recorded from either a GENEActiv or an AX3 device (or a device by other manufacturers if the data have been converted to a standardised HDF5 format and were recorded at 50 Hz or higher and −/+8 g range), tagged with one of three body locations: wrist, lumbar or shin. The data are processed as described Section 2.3 and axes re-labelled to VT, ML and AP orientations. If a lumbar sensor is provided, the pipeline will estimate step/stride lengths and velocity, otherwise only time-based parameters will be extracted. Depending on the body location, the pipeline will deploy a specifically trained model for that sensor location to detect walking bouts. For each bout, the pipeline will proceed to estimate a set of gait events. If the walking bout is longer than 30 s, the software will estimate gait parameters for the entire bout and every 15-second non-overlapping segment, a length suggested as appropriate for gait analysis [34,35]. If there is a period of more than two seconds of non-bout segment detected, the next bout will be a different bout. Additionally, to decrease the presence of false gait events, if two FCs or two ICs are detected with less than a 0.2-second difference, the estimation with higher probability is taken as the right one [15]. 

Once the walking bouts, IC and FC events have been estimated, the software will use these events to estimate time-based parameters, and for the case of the lumbar sensor, IC events are used to segment steps and strides for the step/stride-length DNN models, whereby strides are segmented every two IC events with one-step hops. The pipeline extracts measurements for gait pace, rhythm, variability, symmetry, and postural control. A list of measurements used for our current analysis is shown in Table 1. Supplementary Table S1 shows the full list of core gait parameters estimated by the pipeline. The gait phases (%) in Table 1 are proportions of the gait cycle time which equals the stride time.

### 2.7. Cross-Validation and Fine-Tuning

We implemented a leave-one-subject-out (LOSO) cross-validation procedure to test the performance of the pipeline when encountering unseen data. For this test, we did not differentiate between left or right sides for the case of shin and wrist sensors. For the LOSO validation of the wrist-sensor models, both sensors from the same participant were not included in the training set to prevent data leakage. Fine-tuning of the IC, FC and bout models was carried out by unfreezing all layers of the original network, and the non-walking data from the original multiactivity study (sitting still, pronation-supination, drawing, and front-arm raise) was re-introduced to prevent the DNNs from forgetting non-walking segment detection.

The step/stride length model validation also followed the same LOSO procedure, i.e., the step and stride length models during the LOSO procedure had as input, time series segmented with IC events estimated from the counterpart LOSO U-Net model. The left-out participant was used instead as a validation set for an early stopping TensorFlow-Keras callback with a patience of five epochs and delta = 0.001 [30]. All model training histories were saved, and the median number of epochs (after subtracting patience) stored as a hyperparameter to tune a final model with the full dataset. Results from this final model are not presented here since we did not have (at the time of preparing this manuscript) an independent dataset for its validation. We will, however, test this model on a dataset from an ongoing clinical study in Parkinson’s disease, as part of our future research work [36]. Hence, all the results in the current manuscript are based on the outputs of the LOSO cross-validation procedure only.

### 2.8. Statistical Analysis

Normative gait measurements from the sensing walkway were extracted using the PKMAS software. Mean values per task (slow, normal, fast gait, and TUG) were used for analysis and a diagnostic sensitivity test investigated with a two-way ANOVA for the factors of diagnosis (PD, HC) and walking task (slow, normal, fast gait). The TUG was not included in the diagnostic sensitivity test because it is executed differently from the other three tasks. However, the recorded steps from the TUG were used in all other analyses. For the ANOVA test, we were interested in significance of the diagnosis factor, no interactions were analysed. Posterior Mann–Whitney U tests were implemented post hoc to find which variables drive the differences.

Instrumented gait measurements from the walkway system and our pipeline for the three sensor locations were compared with intraclass correlation coefficient for a two-way random effects, absolute agreement and average rater design, ICC(2,k) [37,38], and Pearson correlation. For interpretation of the ICC values, we used the definitions by Koo and Li [37]: poor (<0.5), moderate (0.5 to <0.75), good (0.75 to 0.9), and excellent (>0.9). For the estimation of ICC values, results from the four gait tasks were collated in a single trial. Visualisation was carried out with Bland–Altman and linear regression plots. To test the sensitivity of the inferred measurements by our pipeline on differentiating PD from HC, we repeated the two-way ANOVA test with Mann–Whitney U tests on the inferred parameters for the three sensor locations. The statistical analysis comprised the parameters shown in Table 1, which included the gait phases (%) for comparison with nominal gait phase proportions.

In order to fairly compare gait measurements from both systems (pressure-sensing walkway and wearable sensors), we masked estimations by the software for each system to only perform event detection during the time the participant was walking on the walkway. The walking tasks were varied and close to free living scenarios; walks included bout initiation and termination steps as well as turning steps; these and all complete steps identified by the walkway system were included in our analysis. Statistical analyses were performed with Python libraries Statsmodels [39] and Pingouin [38].

## 3. Results

For the database in this investigation, one lumbar recording file on one of the PD participants had incomplete data, and the participant was excluded from the analysis since accelerometry data from this location were crucial to visually inspect sensor synchronisation with the walkway system. Two shin and two wrist recordings in the PD group also had incomplete data and were excluded from the analysis. The final database comprised 20 wrist, 11 lumbar, and nine shin accelerometry recordings.

The analysis in this investigation comprises five participants with PD and six HC who had a mean age of 64.2 and 63.4 years old, respectively. PD participants had an average of 5.9 years since clinical diagnosis (minimum 1.5, maximum 9 years), and only the most recently diagnosed participant (1.5 years since diagnosis) was not taking dopaminergic medication. The sex of the participants was not recorded for the pilot study.

### 3.1. Normative Data from the Sensor Walkway

Gait measurements extracted from the pressure-sensing walkway are shown in Table 2. The gait phase parameters of stance %, swing %, and total double support % were statistically significant for the factor of diagnosis (HC vs. PD, *p*-value < 0.05). The Mann–Whitney U tests showed that this significance was mostly driven by differences during the fast gait task (*p*-value = 0.06) for swing % and stance %. Total double support % showed the most important difference during the slow gait task (*p*-value = 0.08).

The gait phases in our study group agreed with normative data for gait cycle phase in older adults: Hollman et al. reported normative gait phase proportions for healthy older adults during normal walking that are close to the values reported in Table 2 [40]. The authors reported a normative stance % between 63.2 and 64.9, a swing % between 35.1 and 36.7, and a double support % between 26.3 and 29.8 [40].

### 3.2. Initial and Final Contact Detection Differences

Differences between matched IC and FC events extracted from the pressure-sensing walkway and our gait pipeline are shown in Table 3, with the values shown in msecs. The mean difference was lower for the lumbar sensor followed by the wrist and shin sensors. However, the subject standard deviation (SSD) and the subject mean absolute value (MAE) were lowest for the lumbar sensor followed by the shin sensor. The wrist sensor showed the largest SSD and MAE values, especially during the fast gait task.

We also explored whether there were differences between groups (PD vs. HC) using multiple Mann–Whitney U tests. For the shin sensor there were significant differences for the slow, normal, and fast gait tasks for both IC and FC events (at a *p*-value < 0.001). For the lumbar sensor, only the slow gait IC and the normal gait FC errors resulted in significant differences. Finally, for the wrist sensor, only the normal and fast gait IC errors showed differences between HC and PD participants.

### 3.3. Estimation of Time-Based Gait Parameters

**Figure 3:**
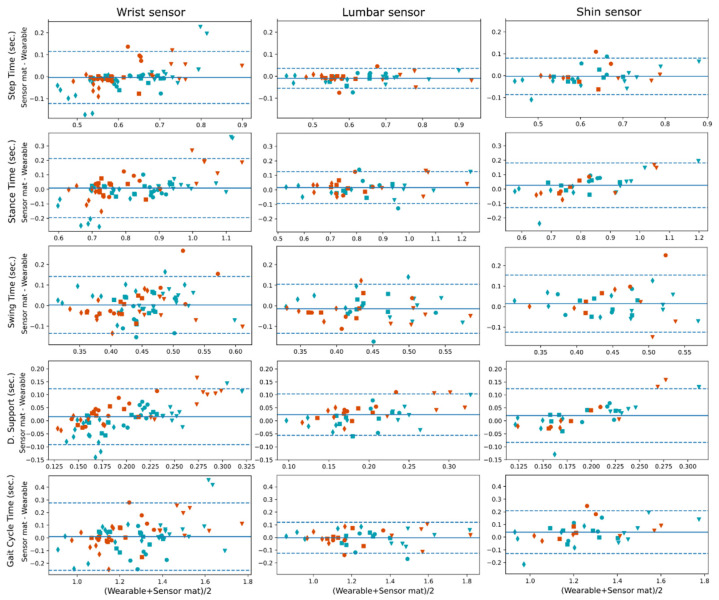
Bland–Altman plots for gait parameter differences between the pressure-sensing walkway and the automatic gait pipeline; mean values per task and shown in seconds. PD participants are plotted in red colour and HC in teal. Slow gait ▼, normal gait ■, fast gait ♦, and timed up and go ●.

We compared gait measurements from both the sensing walkway and the wearable gait pipeline. Figure 3 shows Bland–Altman plots for the mean gait parameter values with the four gait tasks shown within the plots. From the plots, we can see that all sensors showed a negligible bias when compared with the sensing walkway; however, for the double support time, there was a bias when looking at the groups separately, and this was more prominent for the wrist sensor; the wrist sensor tends to estimate shorter double support times for the participants with PD. Regarding the variance, the lumbar sensor showed the highest agreement with the sensing walkway, especially for step time, followed by the shin sensor. The wrist sensor showed the least agreement with the sensing walkway. Bland–Altman plots for stride time, stance %, swing % and double support % are available in the Supplementary Material.

In agreement with the Bland–Altman plots in Figure 3, the ICC coefficients in Table 4 show good-to-excellent agreement values for the lumbar sensor (0.84–1); except for the double support time, which showed a poor coefficient, ICC = 0.37, and the swing time with an ICC = 0.55. The shin sensor also showed good-to-excellent agreement with the pressure-sensing walkway, except for the swing time (ICC = 0.59) and the double support time, ICC = 0.38. The wrist sensor showed moderate (0.5–0.75) to good agreement with the pressure-sensing walkway, except for the swing time and double support time, which showed an ICC(2,k) < 0.60. Specifically, the double support time when estimated with the wrist sensor showed a very poor agreement (ICC = 0.04).

Pearson correlation-based results are also shown in Table 4. All sensors showed good correlations with the pressure-sensing walkway, even the wrist sensor. However, for the shin sensor, Pearson correlation values were slightly lower than for the lumbar sensor. Double support time when estimated with wearable sensors showed significant correlations with the pressure-sensing walkway, despite the poor ICC values. This suggests a bias in the estimation of this parameter from accelerometry data.

### 3.4. Estimation of Space Parameters

**Figure 4:**
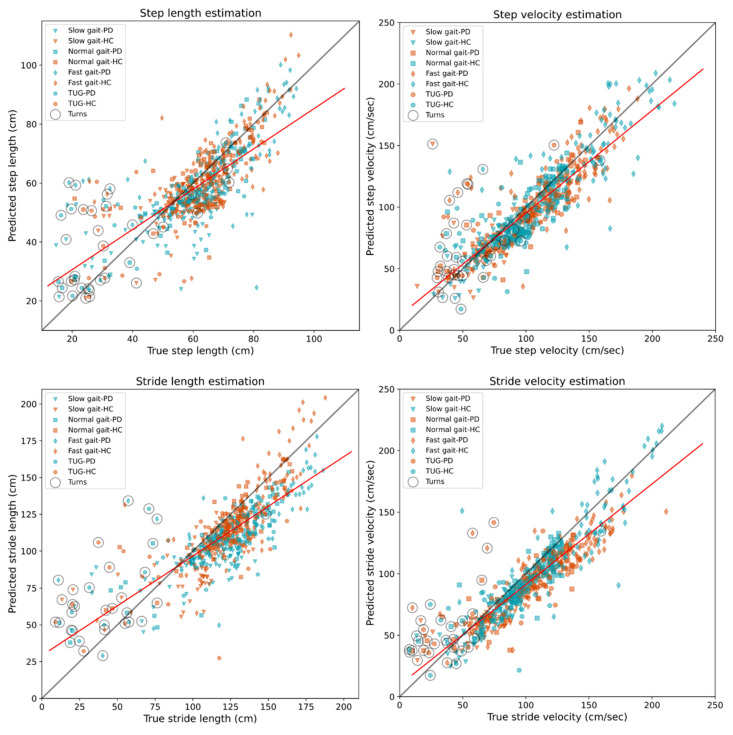
Scatter plot for step and stride length and velocity estimations by the trained models from the LOSO cross-validation. Best linear fit shown with a red line. Participants with PD are shown in red colour and HC in teal. Slow gait ▼, normal gait ■, fast gait ♦, timed up and go ●, turning steps ○.

We were particularly interested in studying the step length and stride length segmentation models, and because of this, we analysed length estimation at the step and stride levels instead of mean values per task, as in the previous section. Figure 4 shows scatter plots for length and velocity estimation, where velocity was computed by dividing the estimated length by the step time. We found a good performance for the step- and stride-length DNN models for all the steps and strides in the database. However, for the turning steps, the DNN algorithm estimated on average longer steps and strides. We identified the turning steps post-analysis by inspecting the feet angles and direction of movement given by the participant’s centre of mass, as estimated by the PKMAS software. These identified turning steps are highlighted within a circle in Figure 4.

ICC and Pearson correlation values for the sensor pipeline compared with the pressure-sensing walkway are given in Table 5. The length and velocity estimations from the sensors showed a good-to-excellent agreement with the pressure-sensing walkway and Pearson correlations coefficients were of >0.75 for the space parameters.

### 3.5. Parameter Diagnostic Sensitivity

To explore the diagnostic sensitivity of the gait measures estimated by the pipeline, we repeated the two-way ANOVA analysis from Section 3.1 for the three sensor locations: lumbar, wrist and shin. For the lumbar sensor, the diagnosis factor was not significant for any of the gait parameters. Despite this, we further explored multiple Mann–Whitney U tests to see if there is a partial agreement with the pressure-sensing walkway. The % parameters were not significant, but the stance time and stride time were significantly different for the normal gait task (*p* = 0.04) when comparing HC vs. PD participants. Consistent with the lumbar sensor, the factor of diagnosis did not reach significance for gait measurements estimated from the shin-based sensor. However, post hoc Mann–Whitney U tests showed significant group differences for double support % (*p* = 0.01), swing % (*p* = 0.04), and stance % (*p* = 0.04) for the normal gait task, suggesting partial agreement with the pressure-sensing walkway results.

For the wrist-estimated parameters, the factor of diagnosis was significant for swing % and the stance % (two-way ANOVA tests, *p* value < 0.026). The Mann–Whitney U tests showed that these variables were not significant but had *p*-values between 0.06 and 0.09 for the three tasks of slow, normal, and fast gait.

## 4. Discussion

In this investigation, we presented a deep learning-based gait analysis pipeline that extracts time and space gait parameters from a single wearable tri-axial accelerometry recording. The sensors can be attached to either the participant’s lower back, shin, or wrist. We trained and validated the DNN models using reference data from a pressure-sensing walkway and compared the performance of our proposed pipeline with the walkway’s extracted gait events and parameters. Shin and lumbar sensors showed very similar performance according to the ICC and Pearson correlation values from core gait parameters. However, MAE and SSD error values in the IC/FC event detection experiments were lower for the lumbar sensor. Overall, our proposed gait analysis pipeline showed fair-to-excellent agreement with the pressure-sensing walkway for the lumbar sensor, followed by the shin and wrist sensors.

Although wrist sensors are not as common as lower limb and waist attached sensors in gait research [41], their superior compliance in free-living clinical studies compared to other body locations demands research with new technologies able to extract valuable clinical parameters. For instance, a study in physical activity by Kerr and colleagues reported a compliance of 98.6% for wrist-worn devices, while hip-worn devices reached only 91.0% of participants (who met the criteria of >600 min wearing time per day) [42]. In large clinical studies, eight percentage points in device-wearing compliance can result in thousands of missing hours of data. In our results, the wrist band sensor presented a bias for the estimation of the double-support time (see Figure 3); wrist bands estimated, on average, shorter double-support times compared to the gold standard in the PD group. This performance bias will be addressed in future research, and strategies to eliminate it could be to gather more data from PD patients and/or focus the AI training on this more challenging group until group biases are negligible. To our knowledge, our study is the first that investigates IC/FC event detection and estimation of derivative parameters from a single wrist-placed sensor device, but more research will be necessary to determine whether its performance will be comparable to that of the lumbar sensor.

As previously mentioned, several algorithms for gait event detection from IMU devices have been proposed. However, despite the current advances it is difficult to directly compare algorithms and results due to the diversity in sensor configurations, e.g., number and location of sensors attached, and data modality used by the algorithms; gyroscope, accelerometer, magnetometer, or combinations of all three. It is reasonable to conclude that increasing the number of sensors—especially for trunk, waist, and lower limbs—and combining gyroscope and accelerometer data, improves gait event detection and precision. Nevertheless, the need to place multiple sensors on a participant makes the gait analysis system impractical for free-living gait; ideally in a clinical study we would like to minimally disrupt participants’ lives. During the development of our gait analysis pipeline, we decided to focus solely on accelerometers to extend the sensor’s battery life by not requiring the use of a gyroscope—a gyroscope consumes on average five times more energy than an accelerometer [12]. We also decided to use a single sensor analysis to minimise participant burden.

McCamley and colleagues reported the performance of three accelerometry-based algorithms with sensors attached to the lower back in a group of healthy young adults [13]. For the authors’ proposed new algorithm, the MAE for IC and FC events were 19 and 32 msecs, respectively. For a second algorithm used as the benchmark, the IC-MAE was 48 msecs and the FC-MAE was 33 msecs. The third algorithm could only estimate ICs with a MAE = 32 msecs. Although these error results are from young adults in a controlled experiment that analysed steady-state gait, the errors reported are within a similar range to our proposed gait analysis pipeline; even our wrist sensor results are not far from those reported by McCamley and colleagues [13]. Hence, we argue that the performance of our proposed analysis pipeline is within an acceptable range for the chosen data modality and sensor configuration.

Another factor that we must consider is the heterogeneity of our gait database, since it included bout step initiations, terminations, and turns. Previous investigations have reported the difficulty of estimating initiation and termination events [21] and specialised algorithms have been designed to detect turns during gait [43]. A common approach to deal with these events is to discard them from the study [44]. This is because most research studies are interested in analysing steady-state walking and not the start or end of a walking bout. In our investigation, we were conscious of the small dataset and decided to keep bout initiations, terminations and turning steps to increase the number of step instances and gait events in our training sets. Additionally, in a free-living or at-home clinical study with older adults, short bouts will comprise most of the collected data [35,45], and will proportionally increase the presence of initiation, termination, and turning steps. Keeping these events in our database gave us an idea of how the algorithm would behave in a free-living study. For instance, we found that estimating step and stride length during turns is particularly challenging, as seen in Figure 4. Additionally, the small percentage of missed steps by our algorithm were mostly step terminations and turns (see Supplementary Material). Future research work will focus on improving detection of these events which are highly common in free-living gait.

Another point to mention, related to the algorithm, is that we did not control for tilted sensors, neither did we subtract the vertical acceleration explicitly. In our investigation, segments were demeaned as part of the signal processing before being band-pass filtered. Despite this, the U-Net algorithm showed to be robust to plausible sensor displacements, and because time series are converted to mel spectrograms, our proposed method for IC and FC event detection is invariant to the sign of the time series. However, it is crucial to have the correct VT, ML, and AP participant’s orientations in relation to the sensor’s axes (despite the positive or negative direction) since the U-Net algorithm is trained with a specific coordinate configuration.

Our implemented automatic pipeline has the potential to extract exploratory parameters for gait complexity and spectral content such as sway, harmonic ratio, dominant frequency, and step/stride regularity; we have implemented a total of 100 gait parameters that can be estimated from the lumbar sensors and 85 from wrist and shin sensors. However, we have yet to validate the accuracy of these exploratory measures. A validation strategy could be to perform a metanalysis comparing our extracted exploratory parameters with results published in the PD literature, and this will be an objective in future research. See Supplementary Material for the full list of parameters estimated.

There is currently a Python library that estimates gait events from a single lower back sensor, GaitPy [11]. GaitPy estimates IC and FC events from vertical accelerometry and uses the Gaussian continuous wavelet transform (CWT) algorithm [13]. A plausible extension of our work could be to include GaitPy’s CWT implementation as a second gait event estimation algorithm for the lumbar sensor and integrate it within our proposed gait analysis pipeline.

## 5. Limitations

Our development has some limitations. One of these limitations is the small sample size of our database; a total of five participants diagnosed with PD and six healthy controls. Our work is a proof-of-concept study for a gait analysis pipeline that infers gait measurements from tri-axial accelerometry. We compared our inferred gait measurements with the ones obtained by a gold standard pressure-sensing walkway, and the data were acquired concurrently. Hence, for the purpose of comparing both technologies and their outcome measures, the small sample size of our pilot study represents a secondary factor that does not affect our conclusions. For the same reason, the reader must be cautious about using our results to compare participant groups (e.g., HC vs. PD comparisons), because the sample size is not large enough for diagnostic conclusions, which were not the aim of our current investigation. Future research work that will focus on generalising our results to a wider population will require a larger representative cohort. 

Data could not be extracted from all recording sessions, meaning that only three shin sensor recordings were available for the PD group. To diminish the impact of a small training dataset, we fine-tuned already trained models from a previous investigation [28]. The previous models were trained with a larger dataset and longer bouts. The model in Peraza et al. [28] was a U-Net that recognises IC events; however, for the FC model in the current investigation we used the same pre-trained model but changed the target from IC to FC events with satisfactory results. Future investigation could focus on using public benchmark databases such as the Parkinson@Home validation study [46], to further analyse performance or tune our U-Net.

Due to the pilot nature of the study, exhaustive clinical and demographic variables were not collected (e.g., the full UPDRS, sex, height, all medications). The pilot study aimed to assess the recruitment and data acquisition protocols, as well as to test the participants’ tolerance to the data acquisition protocols. Learnings from the pilot study were used to design a larger and more comprehensive feasibility study in Parkinson’s disease [36], which was ongoing at the time of preparing this manuscript. As part of our future research, we will test the gait pipeline trained with the full dataset from the current pilot study with the independent sensor data from the feasibility study described in [36].

We aimed to reduce bout segment misclassification by adding multiple non-walking activities during the DNN tuning, but a possibility remains that other types of human activities are misclassified as walking. For instance, Hickey and colleagues reported a participant whose cycling was detected as walking by their implemented algorithm [9]. Further research work will be needed to assess bout misclassification. 

A last point to consider is the use of foot size as a proxy for the participant’s height within the step and stride length models. Previous research in step length estimation has proven the direct relationship between step length and the participant’s height. For instance, the inverted pendulum model uses height for step length estimation from accelerometry [16]. However, in our pilot study, participant’s height was not available and because of this we decided to use foot size as a height proxy. The relationship between height and foot size is well established in the human development and forensic fields [47,48].

## 6. Conclusions

We presented an automatic gait analysis pipeline for wearable sensors that use tri-axial accelerometry and extracts gait parameters from a single sensor attached to either the lower back, wrist, or shin. The detection of gait events is based on deep learning algorithms (U-Nets) and showed a comparable performance with published algorithms as well as good agreement with a gold-standard pressure-sensing walkway. Derivative data supporting this investigation—estimated parameters by the automatic pipeline and measurements extracted by the pressure-sensing walkway—are available upon request. Overall, our results in this investigation show the potential viability for the use of wearable sensors in clinical and research studies for the assessment of gait in free-living and at-home scenarios.

## Figures and Tables

**Table 1:** Gait parameters estimated by the gait pipeline and used in our analyses.

Pace	Rhythm
Step velocity	Step time
Step length	Swing time
Stride length	Stance time
Stride velocity	Stride time
Stance %	Double support time
Swing %	
Double support time %	

**Table 2:** Instrumented gait analysis from the pressure-sensing walkway. Stride time equals the Gait cycle time. * Significant results for the diagnosis factor in a two-way ANOVA test, *p*-value < 0.05.

	Slow Gait		Normal Gait		Fast Gait		TUG		Diagnosis
Mean (SD)	HC	PD	HC	PD	HC	PD	HC	PD	F(1,29) *p*-Value
**Step length (cm)**	54.15 (6.54)	50.6 (7.75)	63.01 (8.20)	62.32 (12.36)	71.69 (6.07)	67.23 (14.86)	52.10 (12.09)	51.15 (5.88)	0.79 0.378
**Step Time (s)**	0.75 (0.08)	0.78 (0.089)	0.611 (0.065)	0.568 (0.030)	0.50 (0.072)	0.517 (0.027)	0.64 (0.056)	0.60 (0.08)	0.002 0.961
**Stance Time (s)**	1.035 (0.14)	1.086 (0.12)	0.806 (0.09)	0.762 (0.042)	0.65 (0.10)	0.67 (0.039)	0.88 (0.021)	0.77 (0.078)	0.06 0.808
**Swing Time (s)**	0.51 (0.043)	0.489 (0.049)	0.44 (0.035)	0.399 (0.049)	0.42 (0.08)	0.37 (0.07)	0.43 (0.06)	0.45 (0.131)	3.08 0.089
**Stride length (cm)**	106.7 (13.43)	98.36 (11.34)	124.4 (17.3)	119.7 (23.6)	135.9 (11.9)	128.3 (20.7)	106.76 (18.62)	104.4 (12.34)	1.49 0.231
**Stride time (s)**	1.54 (0.17)	1.59 (0.15)	1.24 (0.111)	1.15 (0.076)	1.06 (0.167)	1.06 (0.09)	1.29 (0.105)	1.22 (0.15)	0.08 0.774
**Total D. Support (s)**	0.54 (0.10)	0.628 (0.1)	0.37 (0.073)	0.37 (0.062)	0.26 (0.054)	0.31 (0.052)	0.45 (0.05)	0.42 (0.10)	2.59 0.117
**Stance time %**	67.49 (1.91)	69.4 (2.4)	64.84 (1.92)	65.87 (2.55)	61.72 (2.23)	64.78 (2.86)	66.72 (2.55)	64.47 (5.44)	6.45 0.016 *
**Swing time %**	32.5 (1.91)	30.55 (2.40)	35.15 (1.92)	34.12 (2.55)	38.27 (2.23)	35.21 (2.86)	33.24 (2.55)	35.52 (5.44)	6.45 0.016 *
**Total D. Support time %**	34.97 (3.93)	38.8 (4.06)	29.91 (4.10)	32.11 (5.17)	25.17 (3.55)	29.28 (5.59)	33.39 (4.53)	32.2 (4.67)	5.12 0.031 *
**Cadence (steps/min)**	77.39 (8.0)	74.76 (7.2)	96.08 (8.33)	103.2 (6.01)	114.9 (16.3)	110.09 (9.01)	93.89 (7.98)	95.74 (12.64)	0.001 0.974
**Velocity (cm/s)**	69.57 (10.06)	61.93 (9.5)	100.2 (13.0)	103.8 (20.64)	130.32 (20.47)	121.04 (15.89)	82.70 (10.76)	86.31 (12.36)	0.64 0.414

**Table 3:** Initial contact (IC) and final contact (FC) differences between the pressure-sensing walkway and wearable sensor estimations. Values shown in milliseconds. Mean is the arithmetic mean, SSD stands for subject standard deviation, and MAE for subject mean absolute error.

Location		Slow Gait		Normal Gait		Fast Gait		TUG	
Lumbar		HC	PD	HC	PD	HC	PD	HC	PD
**Initial Contact**	Mean:	−8.95	8.21	12.38	9.59	7.48	13.72	2.01	−11.05
	SSD:	31.97	38.84	23.62	39.19	31.32	33.14	34.79	37.62
	MAE:	31.83	27.35	22.82	29.16	33.09	29.26	30.73	32.03
**Final Contact**	Mean:	6.96	15.73	4.62	21.02	−4.25	13.40	−5.22	−2.82
	SSD:	45.06	46.48	31.29	29.06	35.89	48.42	34.59	39.1
	MAE:	50.31	39.76	31.22	32.64	31.53	31.01	35.46	30.61
**Wrist**									
**Initial Contact**	Mean:	7.89	−6.85	25.0	−1.95	10.87	−14.5	22.28	−15.76
	SSD:	55.34	74.03	39.24	48.98	88.23	75.64	61.47	46.3
	MAE:	55.33	62.55	40.19	45.38	72.70	68.57	56.19	50.8
**Final Contact**	Mean:	4.91	−10.31	21.61	5.58	15.94	−20.92	16.55	−10.9
	SSD:	72.57	90.0	42.18	42.23	88.39	72.16	52.1	51.6
	MAE:	59.38	78.7	49.97	46.03	74.01	72.87	49.31	49.47
**Shin**									
**Initial Contact**	Mean:	−1.43	−41.82	21.16	−13.79	18.57	−13.04	5.029	−7.29
	SSD:	39.97	67.45	25.63	37.97	39.26	30.55	52.12	53.21
	MAE:	42.38	64.66	28.56	40.05	34.5	33.54	39.15	45.67
**Final Contact**	Mean:	32.86	−18.43	28.58	−17.60	12.74	−36.57	14.06	−0.04
	SSD:	44.74	71.2	35.21	27.63	54.27	53.63	43.96	71.03
	MAE:	55.84	60.18	47.82	36.06	44.83	51.98	42.75	57.99

**Table 4:** Intraclass correlation coefficients (ICC) and Pearson correlation results for comparison between the pressure-sensing walkway and the wearable sensor pipeline.

Sensor	Parameter	ICC(2,k)	Pearson ρ
**Lumbar**	Step time	0.97 *p* < 0.001	0.98 *p* < 0.001
	Stride time	0.92 *p* < 0.001	0.97 *p* < 0.001
	Stance time	0.91 *p* < 0.001	0.94 *p* < 0.001
	Swing time	0.55 *p* = 0.15	0.63 *p* < 0.001
	D. support time	0.37 *p* = 0.20	0.80 *p* < 0.001
	Gait cycle time	0.91 *p* < 0.001	0.96 *p* < 0.001
	Cadence	0.84 *p* = 0.001	0.96 *p* < 0.001
**Wrist**	Step time	0.85 *p* = 0.004	0.85 *p* < 0.001
	Stride time	0.83 *p* = 0.004	0.84 *p* < 0.001
	Stance time	0.69 *p* = 0.04	0.81 *p* < 0.001
	Swing time	0.58 *p* = 0.10	0.48 *p* < 0.001
	D. support time	0.04 *p* = 0.47	0.58 *p* < 0.001
	Gait cycle time	0.82 *p* = 0.003	0.79 *p* < 0.001
	Cadence	0.76 *p* = 0.002	0.83 *p* < 0.001
**Shin**	Step time	0.98 *p* < 0.001	0.92 *p* < 0.001
	Stride time	0.97 *p* < 0.001	0.92 *p* < 0.001
	Stance time	0.93 *p* < 0.001	0.91 *p* < 0.001
	Swing time	0.59 *p* = 0.08	0.49 *p* = 0.003
	D. support time	0.38 *p* = 0.15	0.66 *p* < 0.001
	Gait cycle time	0.95 *p* < 0.001	0.92 *p* < 0.001
	Cadence	0.92 *p* < 0.001	0.90 *p* < 0.001

**Table 5:** Intraclass correlation coefficient (ICC) and Pearson correlations between pressure-sensing walkway and the gait analysis pipeline for space parameters.

Parameter	ICC(2,k)	Pearson ρ
Step length	0.93 *p* < 0.001	0.753 *p* < 0.001
Step velocity	0.92 *p* < 0.001	0.861 *p* < 0.001
Stride length	0.79 *p* < 0.001	0.792 *p* < 0.001
Stride velocity	0.82 *p* < 0.001	0.892 *p* < 0.001

## Data Availability

Derivative data that support the findings in this investigation (pressure-sensing walkway and gait pipeline estimated measurements) are available from IXICO (L.R.P.). Sensor and clinical data are not publicly available.

## Supplementary Materials

The following are available online at https://www.mdpi.com/article/10.3390/s21248286/s1, Table S1: Gait parameters estimated by the pipeline implementation. Table S2: ICC values and Pearson correlation for gait phase estimations. Figure S1: Bland–Altman plots for the remainder of parameters not shown in Figure 3. Figures S2 and S3: Gait events missed from sensors for all participants and tasks. Figure S4: Supplementary. Gait events missed from the wrist sensor for all participants and tasks. Initial contacts are shown in orange and final contacts are shown in teal color. Table S3: Full list of pipeline-estimated parameters by sensor location.
