# Supplementary material for: An Automatic Gait Analysis Pipeline for Wearable Sensors: A Pilot Study in Parkinson’s Disease
Source: Sensors (Basel). 2021 Dec 11;21(24):8286. doi: 10.3390/s21248286 (PMC8707484; doi:10.3390/s21248286)
Supplement: Supplementary file 1 [file sensors-21-08286-s001.zip › sensors-1445112-supplementary.pdf]

Supplementary Material

An Automatic Gait Analysis Pipeline for Wearable Sensors: A Pilot Study in Parkinson's Disease

Luis R. Peraza [1], Kirsi M. Kinnunen [1], Roisin McNaney [2], Ian J. Craddock [3], Alan L. Whone [4,5], Catherine Morgan [4,5,*], Richard Joules [1] and Robin Wolz [1,6]

[1] IXICO, EC1A 9PN London, UK; luis.peraza@ixico.com (L.R.P.); kirsi.kinnunen@ixico.com (K.M.K.); richard.joules@ixico.com (R.J.); robin.wolz@ixico.com (R.W.)
[2] Monash University, Department of Human Centred Computing, Australia; roisin.mcnaney@monash.edu
[3] University of Bristol, School of Computer Science, Electrical and Electronic Engineering, BS8 1QU Bristol, UK; ian.craddock@bristol.ac.uk
[4] University of Bristol Medical School, Translational Health Sciences, BS8 1QU Bristol, UK; alan.whone@bristol.ac.uk
[5] North Bristol NHS Trust, Movement Disorders Group, Westbury on Trym, BS10 5NB Bristol, UK
[6] Department of Computing, Imperial College London, SW7 2AZ London, UK
* Correspondence: catherine.morgan@bristol.ac.uk

Citation: Peraza, L.R.; Kinnunen, K.M.; McNaney, R.; Craddock, I.J.; Whone, A.L.; Morgan, C.; Joules, R.; Wolz, R. An Automatic Gait Analysis Pipeline for Wearable Sensors: A Pilot Study in Parkinson's Disease. *Sensors* **2021**, *21*, 8286. https://doi.org/10.3390/s21248286

Academic Editors: Barry Greene; Rahul Soangra; Alan Bourke

Received: 16 October 2021
Accepted: 8 December 2021
Published: 11 December 2021

Publisher's Note: MDPI stays neutral with regard to jurisdictional claims in published maps and institutional affiliations.

Copyright: © 2021 by the authors. Submitted for possible open access publication under the terms and conditions of the Creative Commons Attribution (CC BY) license (https://creativecommons.org/licenses/by/4.0/).

1. Core gait measurements estimated by the pipeline

Table S1. supplementary. Gait parameters estimated by the pipeline implementation. Standard deviation (SD), Absolute symmetry index (ASI).

Pace	Rhythm	Variability	Asymmetry	Postural control
Step velocity	Step time	Step time SD	Step time ASI	Step length ASI
Step length	Swing time	Stance time SD	Swing time ASI	
Swing time SD	Stance time	Step velocity SD	Stance time ASI	
Stride length	Stride time	Step length SD	Stride time ASI	
Stride velocity	Double support time	Stride time SD		
Stance %		Swing time SD		
Swing %		Double support time SD		
Double support time %				

Standard deviation (SD) is estimated within bout or sub-bout segments, and the absolute symmetry index (ASI) is estimated by taking the even and odd steps/strides separately within a bout.

2. Bland-Altman plots and ICC values.

Figure S1 shows Bland-Altman plots for the remainder of endpoints that were not shown in Figure 3 in the main document. Table S2 shows the intraclass correlation coefficients, ICC(2,1), for the gait phases of swing %, stance %, and double support %. The gait phase endpoints showed very poor agreement with their equivalents extracted from the force platform. The poor agreement was due to the high variance and the narrow range of values: swing % ~ 35, stance % ~65, double support % ~ 15. However, despite the high variance the estimated gait phases showed a negligible bias (as shown by the Bland-Altman plots).

Table S2. supplementary. Intraclass correlation coefficient (ICC) and Pearson correlation for the gait phase estimations.

Sensor	Endpoint	ICC(2,1)	Pearson ρ
**Lumbar**	Swing %	0.0 p=0.67	0.23 p=0.13
	Stance %	0.0 p=0.66	0.19 p=0.23
	D. Support %	0.0 p=0.59	0.32 p=0.04
**Wrist**	Swing %	0.12 p=0.42	0.14 p=0.21
	Stance %	0.0 p=0.63	-0.024 p=0.83
	D. Support %	0.10 p=0.42	-0.10 p=0.35
**Shin**	Swing %	0.38 p=0.26	0.32 p=0.07
	Stance %	0.0 p=0.66	-0.20 p=0.24
	D. Support %	0.0 p=0.84	-0.012 p=0.94

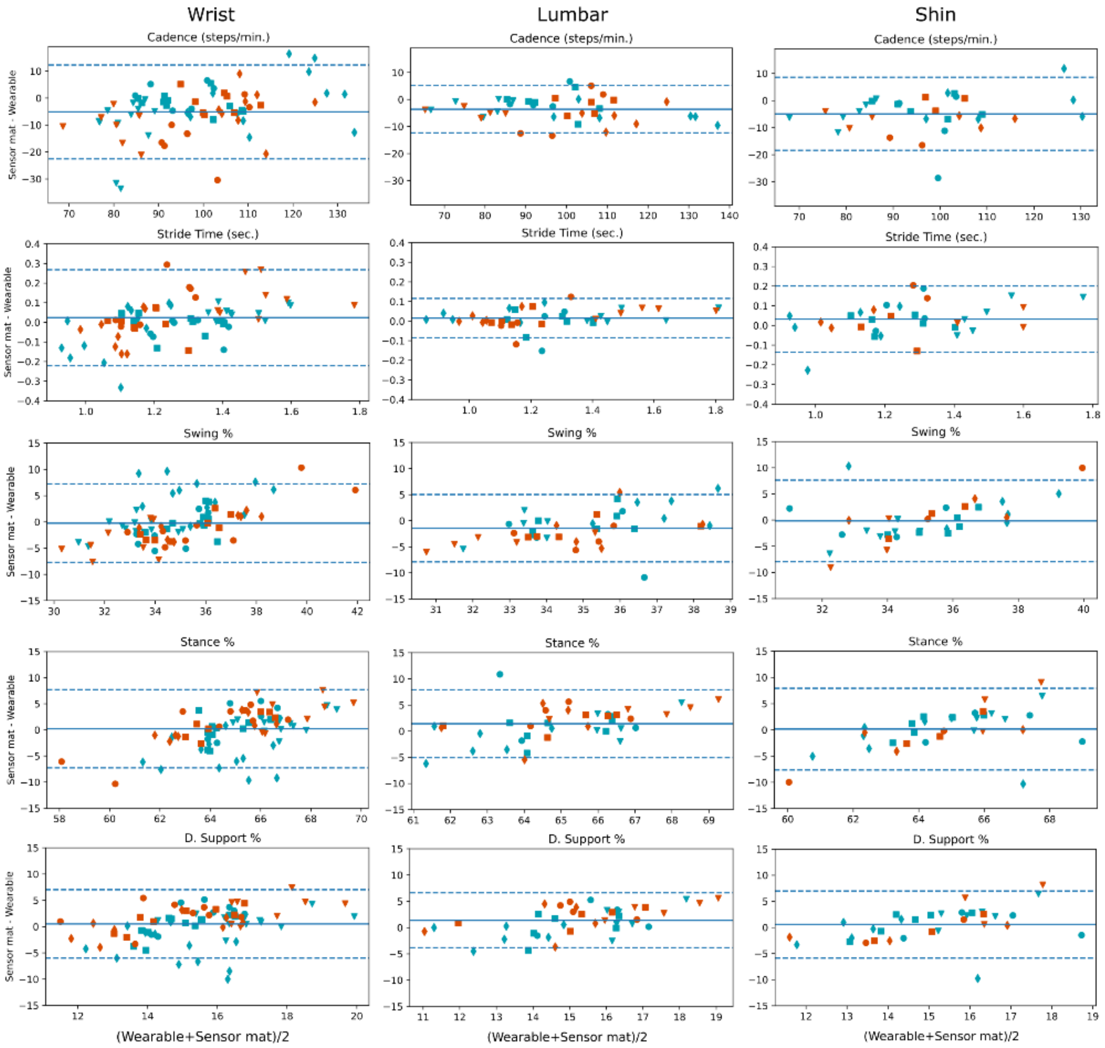

Figure S1. Supplementary. Bland-Altman plots for the remainder of endpoints not shown in main-document Figure 3.

3. Matching steps between accelerometers and the force platform

Gait events, initial contact (IC) and final contact (FC), were matched between the sensor devices and the force platform by a time length criterion of 0.4 seconds; if there was a separation between the closest pipeline-estimated event and the reference platform event of more than 400 milliseconds (msecs), the event was considered missed or non-detected. Figures S3, S4 and S5 show missed gait events for the lumbar, shin and wrist sensors respectively. Because participants performed variable number of steps while on the platform, we normalised the participants' events to a fixed-length and normalized the number of events in a walking event list. We then multiplied the array by the total number of missed events. This allowed us to aggregate—for visualisation—the missed event arrays from all participants, for each gait task and wearable sensor.

Participants in the study performed between two to four walks on the force platform. For instance, for the slow-gait task, the participants did four walks (including three turns) and for the timed up and go (TUG) task participants did two walks, which included a single turn. The IC and FC U-Net models showed difficulty in estimating events from turns and from bout termination steps (see Figure 2).

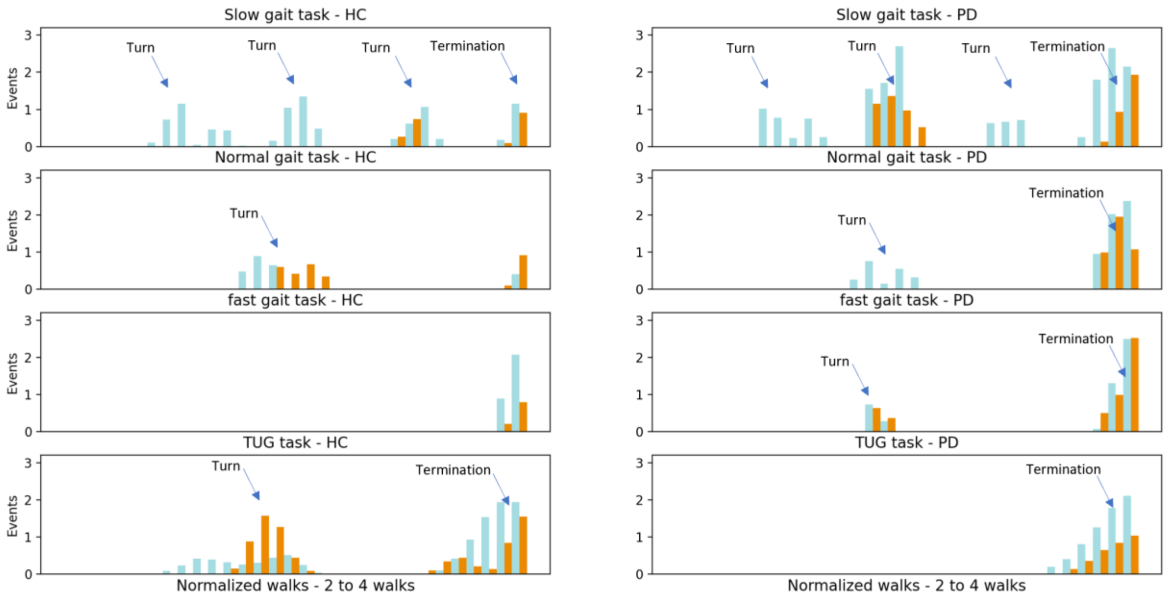

Figure S2. Gait events missed from the **lumbar sensor** for all participants and tasks. Initial contacts are shown in orange and final contacts in teal color.

Notice as well that during the TUG task all wearable sensors showed difficulty in estimating events at the bout termination. We hypothesize that this happened due to the body movements performed by the participants when preparing to again sit down on the chair.

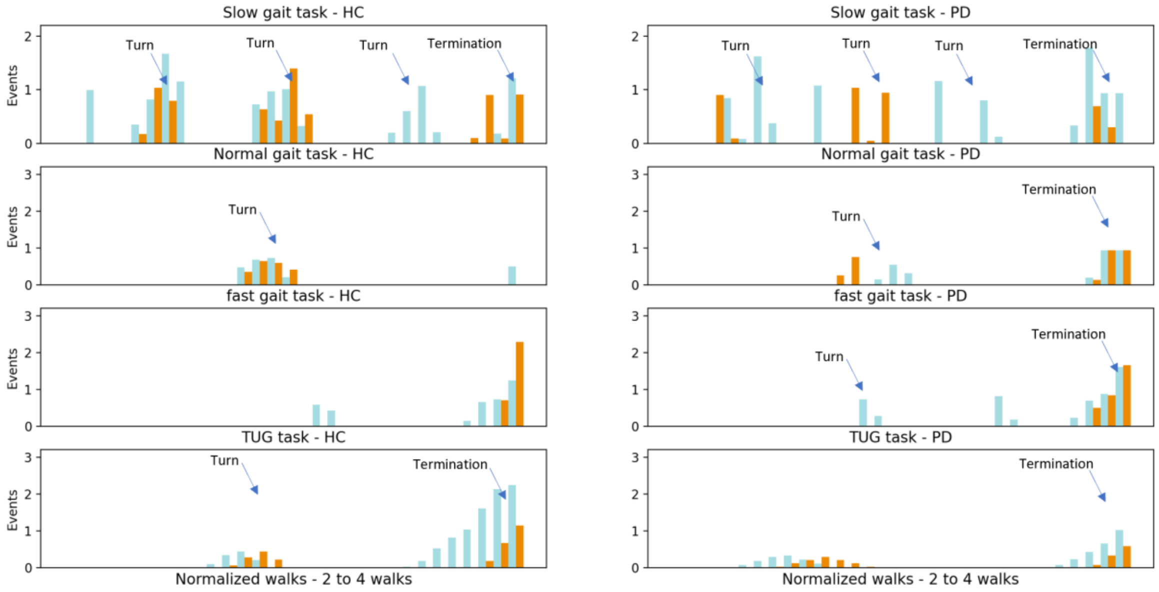

Figure S3. Gait events missed from the **shin sensor** for all participants and tasks. Initial contacts are shown in orange and final contacts are shown in teal color.

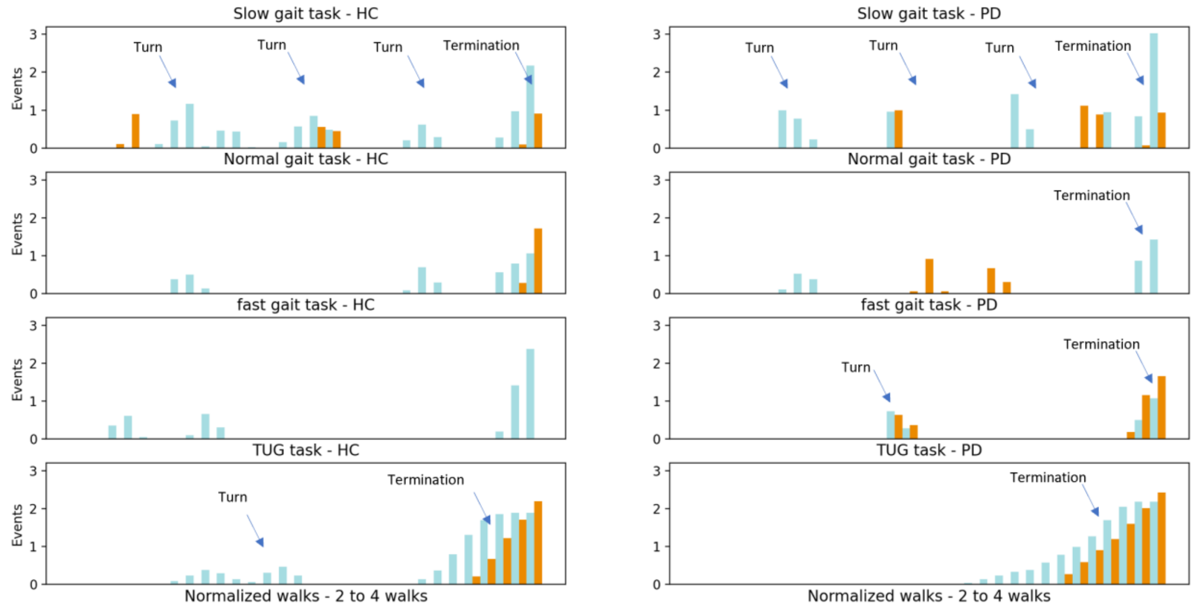

Figure S4. Supplementary. Gait events missed from the **wrist sensor** for all participants and tasks. Initial contacts are shown in orange and final contacts are shown in teal color.

4. Full list of gait endpoints estimated by the automatic pipeline

Table S3 shows the full list of endpoints estimated by the automatic pipeline from triaxial accelerometry data. For a lumbar sensor, 100 endpoints are estimated while for the shin and wrist sensors 85 endpoints are estimated. This is because no space metrics (velocity and length) are extracted from the wrist and shin locations. Space metrics are only estimated for the lumbar sensor.

Global endpoints can be estimated per day or for the entirety of the recording. These are considered measures of macrogait. The rest of the endpoints for microgait are estimated per bout and sub-bouts, and include measures of pace, rhythm, variability, symmetry, and postural control, as well as measures of gait complexity and spectral content.

Table S3. Full list of estimated endpoints by sensor location. 1=yes, 0=no.

	Lumbar	*Wrist/Shin*
Global endpoints (Macro-gait)		
Cadence	1	1
Gait velocity	1	0
Step count	1	1
Number of bouts	1	1
Walking volume	1	1
Longest bout	1	1
Bout time (mean)	1	1
Bout time SD	1	1
Step velocity 50th percentile	1	0
Step velocity 95th percentile	1	0
Stride velocity 50th percentile	1	0

Stride velocity 95th percentile	1	0
Step length 50th percentile	1	0
Step length 95th percentile	1	0
Stride length 50th percentile	1	0
Stride length 95th percentile	1	0
Stance time 50th percentile	1	1
Stance time 95th percentile	1	1
Swing time 50th percentile	1	1
Swing time 95th percentile	1	1
Micro-gait endpoints		
Bout time	1	1
Number of steps	1	1
Range g ML -min	1	1
Range g ML -mean	1	1
Range g ML -max	1	1
Range g AP -min	1	1
Range g AP -mean	1	1
Range g AP -max	1	1
Range g VT -min	1	1
Range g VT -mean	1	1
Range g VT -max	1	1
Pace		
Step velocity	1	0
Step length	1	0
Swing time SD	1	1
Stride length	1	0
Stride velocity	1	0
Stance %	1	1
Swing %	1	1
Double support %	1	1
Rhythm		
Step time	1	1
Swing time	1	1
Stance time	1	1
Stride time	1	1
Double support time	1	1
Variability		
Step time SD	1	1
Stance time SD	1	1
Step velocity SD	1	1
Step length SD	1	0
Stride time SD	1	1
Swing time SD	1	1

Double support SD	1	1
Symmetry		
Step time ASI	1	1
Swing time ASI	1	1
Stance time ASI	1	1
Stride time ASI	1	1
Postural control		
Step length ASI	1	0
Step width	0	0
Complexity endpoints		
Harmonic ratio ML	1	1
Harmonic ratio AP	1	1
Harmonic ratio VT	1	1
Sway Jerkiness ML	1	1
Sway Jerkiness AP	1	1
Sway Jerkiness VT	1	1
JERK (Sway ML+PA)/2	1	1
Power (total freq) ML	1	1
Power (total freq) AP	1	1
Power (total freq) VT	1	1
F50 ML	1	1
F50 AP	1	1
F50 VT	1	1
F95 ML	1	1
F95 AP	1	1
F95 VT	1	1
Dominant frequency (DF) ML	1	1
DF AP	1	1
DF ML	1	1
DF width ML	1	1
DF width AP	1	1
DF width VT	1	1
DF amplitude ML	1	1
DF amplitude AP	1	1
DF amplitude VT	1	1
Dominant peaks # AP	1	1
Dominant peaks # VT	1	1
Low freq % <10 Hz ML	1	1
Low freq % <0.7 Hz AP	1	1
Root mean square (RMS) ML	1	1
RMS AP	1	1
RMS VT	1	1
Step regularity ML	1	1

Step regularity AP	1	1
Step regularity VT	1	1
Stride regularity ML	1	1
Stride regularity AP	1	1
Stride regularity VT	1	1
Spectral centroid ML	1	1
Spectral centroid AP	1	1
Spectral centroid VT	1	1
Max Lyapunov exponent ML	1	1
Max Lyapunov exponent AP	1	1
Max Lyapunov exponent VT	1	1

1. Sensor positioning

Axivity AX3

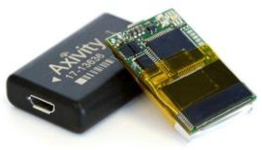

Axivity AX3

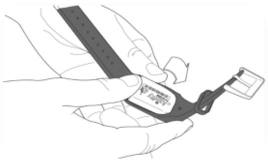

For positioning on the wrist, a wrist band is used. This has a convention where the arrow on the AX3 aligns with the arrow on the band.

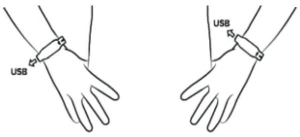

With the right wrist, the USB port is configured to point towards the ground. With the left wrist, the USB port should point up.

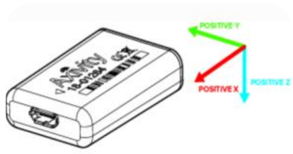

When the AX3 is mounted on the lower back (over L5 - lumbar spine), the USB port should point towards the ground. The side with the text should be against the back.

From: https://axivity.com/files/resources/AX3-User-Guide-v1-2.pdf

GENEActiv Original

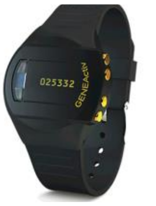

GENEActiv Original

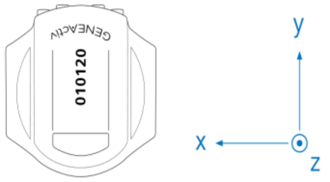

On the right hand, with the arm relaxed to the side, the device will appear like this to the observer.

.

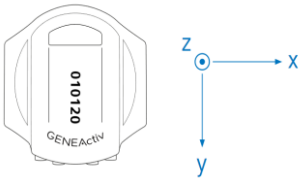

On the left hand, with the arm relaxed to the side, the device will appear like this to the observer.

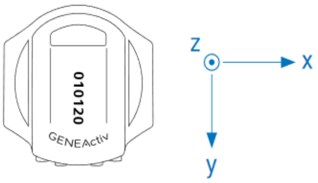

When worn on the shin, the device should be fitted with gold contact pins towards the ground.

From: https://49wvycy00mv416l561vrj345-wpengine.netdna-ssl.com/wp-content/uploads/2014/03/geneactiv_instruction_manual_v1.2.pdf
